# Supplementary material for: Prediction of Klebsiella phage-host specificity at the strain level
Source: Nat Commun. 2024 May 22;15:4355. doi: 10.1038/s41467-024-48675-6 (PMC11111740; doi:10.1038/s41467-024-48675-6)
Supplement: Supplementary file 1 — Supplementary Information [file 41467_2024_48675_MOESM1_ESM.pdf]

Prediction of *Klebsiella* phage-host specificity at the strain level

Dimitri Boeckaerts[1,2], Michiel Stock[2], Celia Ferriol-González[3], Jesús Oteo-Iglesias[4,5], Rafael Sanjuán[3], Pilar Domingo-Calap[3], Bernard De Baets[2], Yves Briers[1,*]

Supplementary Figure S1

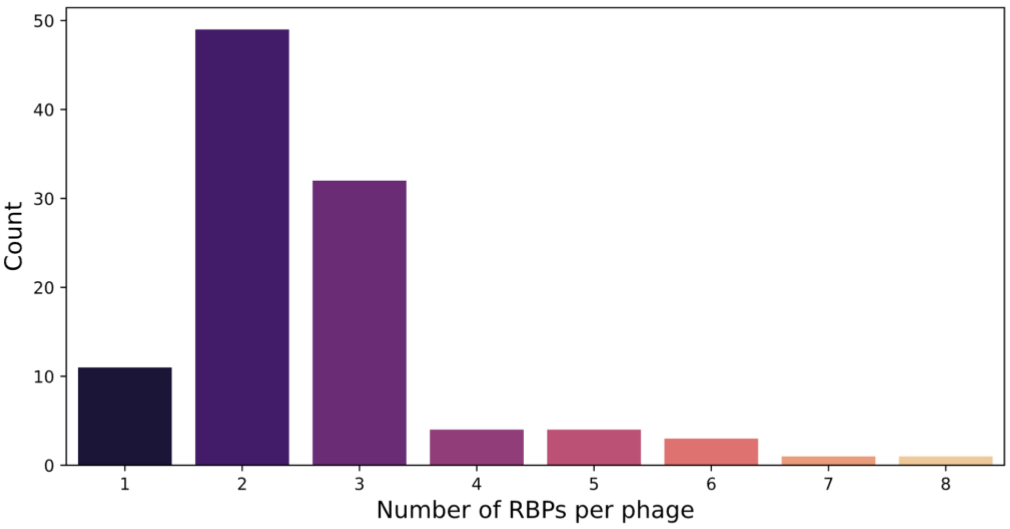

Figure S1: Count plot of the number of detected RBPs per phage across all the collected phage genomes (color scaled from low to high numbers).

Supplementary Figure S2

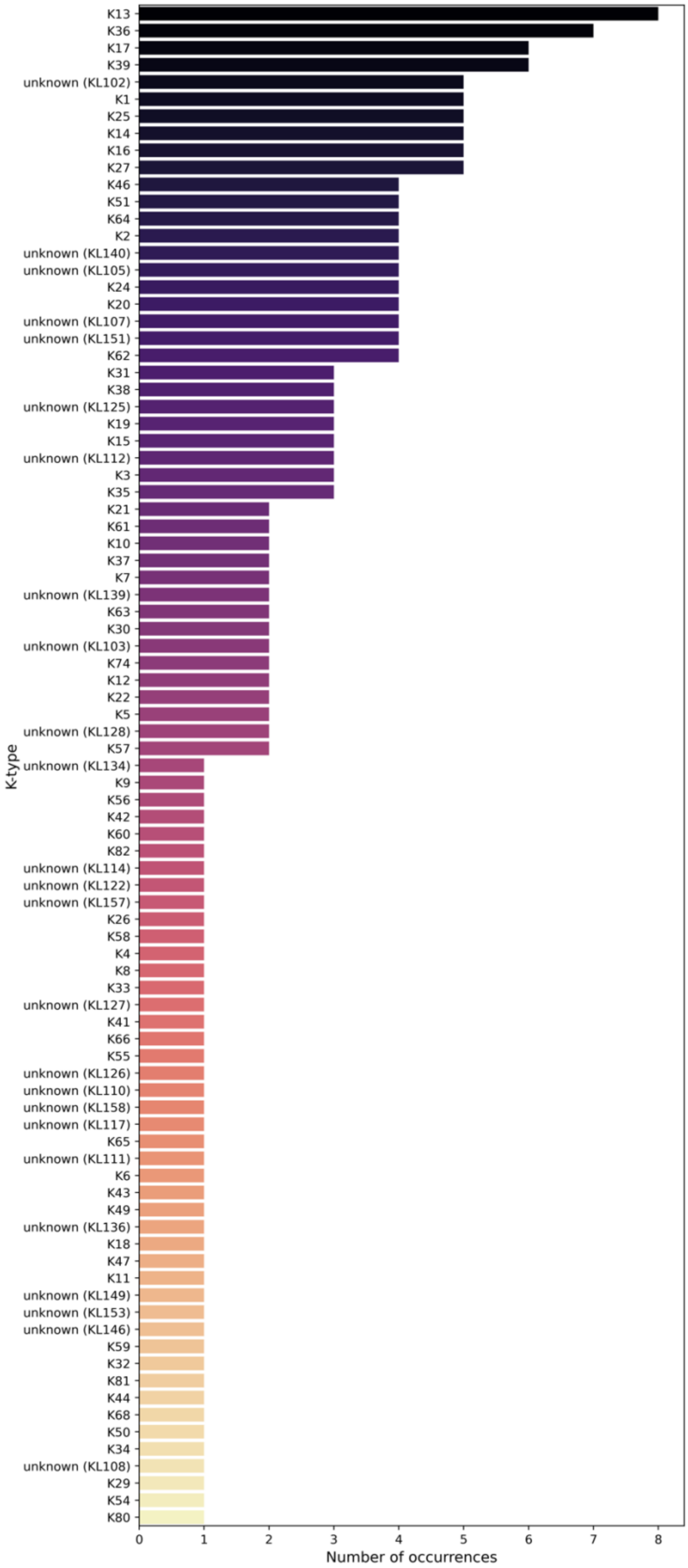

Figure S2: Count plot of the number of occurrences of each K-type across the 200 collected bacterial genomes (color scaled from high to low numbers).
