# Supplementary material for: Prediction of Klebsiella phage-host specificity at the strain level
Source: Nat Commun. 2024 May 22;15:4355. doi: 10.1038/s41467-024-48675-6 (PMC11111740; doi:10.1038/s41467-024-48675-6)
Supplement: Supplementary file 3 — Description of Additional Supplementary Files [file 41467_2024_48675_MOESM3_ESM.pdf]

Description of Additional Supplementary Files

Supplementary Dataset 1. Top-five ranked phage candidates as predicted by PhageHostLearn for each of the 28 *Klebsiella* clinical isolates.

Supplementary Dataset 2: Laboratory confirmations of all of the 476 tested phage-host interactions with spot tests. Numbered cells reflect a confirmed interaction: -3 means the interaction was confirmed both at 10^{-1} and 10^{-3} dilutions; -1 means the interaction was only confirmed at a 10^{-1} dilution, always in duplicate or triplicate. Blank cells reflect interactions that could not be confirmed.

Supplementary Dataset 3: Performance metrics of the *in vitro* validation, including the top-five hit ratio, the percentage of correct predictions, the true positives, the false positives and the false negatives.

Supplementary Dataset 4: Detailed look at the true positives, false positives and false negatives in the top-five predictions of the *in vitro* validation.

Supplementary Dataset 5: Identified antiphage defense systems in the 28 clinical isolates of the *in vitro* validation using DefenseFinder.
