# Supplementary material for: Prediction of Klebsiella phage-host specificity at the strain level
Source: Nat Commun. 2024 May 22;15:4355. doi: 10.1038/s41467-024-48675-6 (PMC11111740; doi:10.1038/s41467-024-48675-6)
Supplement: Supplementary file 5 — Reporting Summary [file 41467_2024_48675_MOESM5_ESM.pdf]

nature portfolio

Corresponding author(s): Yves Briers

Last updated by author(s): Apr 29, 2024

Reporting Summary

Nature Portfolio wishes to improve the reproducibility of the work that we publish. This form provides structure for consistency and transparency in reporting. For further information on Nature Portfolio policies, see our Editorial Policies and the Editorial Policy Checklist.

Statistics

For all statistical analyses, confirm that the following items are present in the figure legend, table legend, main text, or Methods section.

n/a	Confirmed	
☐	☒	The exact sample size (*n*) for each experimental group/condition, given as a discrete number and unit of measurement
☐	☒	A statement on whether measurements were taken from distinct samples or whether the same sample was measured repeatedly
☒	☐	The statistical test(s) used AND whether they are one- or two-sided<br>*Only common tests should be described solely by name; describe more complex techniques in the Methods section.*
☐	☒	A description of all covariates tested
☒	☐	A description of any assumptions or corrections, such as tests of normality and adjustment for multiple comparisons
☐	☒	A full description of the statistical parameters including central tendency (e.g. means) or other basic estimates (e.g. regression coefficient) AND variation (e.g. standard deviation) or associated estimates of uncertainty (e.g. confidence intervals)
☒	☐	For null hypothesis testing, the test statistic (e.g. *F*, *t*, *r*) with confidence intervals, effect sizes, degrees of freedom and *P* value noted<br>*Give P values as exact values whenever suitable.*
☒	☐	For Bayesian analysis, information on the choice of priors and Markov chain Monte Carlo settings
☒	☐	For hierarchical and complex designs, identification of the appropriate level for tests and full reporting of outcomes
☒	☐	Estimates of effect sizes (e.g. Cohen's *d*, Pearson's *r*), indicating how they were calculated

Our web collection on statistics for biologists contains articles on many of the points above.

Software and code

Policy information about availability of computer code

Data collection	No software was used to collect raw data.
Data analysis	We provide full access to our code through GitHub (https://github.com/dimiboeckaerts/PhageHostLearn) and Zenodo (https://doi.org/10.5281/zenodo.11074747). Sequence data were processed using PHANOTATE v1.5.0 (https://github.com/deprekate/PHANOTATE), PhageRBPdetection v2.1.3 (https://github.com/dimiboeckaerts/PhageRBPdetection) and Kaptive v2.0.0 (https://github.com/klebgenomics/Kaptive). Feature representations of processed sequences were computed using ESM-2 v1.0.3 (https://github.com/facebookresearch/esm). The machine learning model used XGBoost v1.5.0 (https://github.com/dmlc/xgboost) and we evaluated the model using cross-validation and metrics implemented in Scikit-learn v0.24.2 (https://scikit-learn.org/stable/). Furthermore, our code pipeline uses python v3.9.7, biopython v1.79, joblib v1.1.0, json v4.2.1, matplotlib v3.4.3, numpy v1.20.3, pandas v1.3.4, pickle 0.7.5 and seaborn v0.11.2.

For manuscripts utilizing custom algorithms or software that are central to the research but not yet described in published literature, software must be made available to editors and reviewers. We strongly encourage code deposition in a community repository (e.g. GitHub). See the Nature Portfolio guidelines for submitting code & software for further information.

Data

Policy information about availability of data

All manuscripts must include a data availability statement. This statement should provide the following information, where applicable:

- Accession codes, unique identifiers, or web links for publicly available datasets
- A description of any restrictions on data availability
- For clinical datasets or third party data, please ensure that the statement adheres to our policy

Genome sequence data were collected in FASTA format from Beamud et al. (Cell Reports, 2023) and from Ferriol-Gonzalez et al. (Biorxiv, 2024); both associated to the Intitute for Integrative Systems Biology (I2SysBio) in Spain. We provide full availability to (1) all collected raw sequence data collected in FASTA format; (2) the processed data that were used in the analyses and to train and evaluate the machine learning model and (3) the phage-host interaction data in a .csv format. These data are available through Zenodo (https://doi.org/10.5281/zenodo.8095914).

Research involving human participants, their data, or biological material

Policy information about studies with human participants or human data. See also policy information about sex, gender (identity/presentation), and sexual orientation and race, ethnicity and racism.

Reporting on sex and gender	N/A
Reporting on race, ethnicity, or other socially relevant groupings	N/A
Population characteristics	N/A
Recruitment	N/A
Ethics oversight	N/A

Note that full information on the approval of the study protocol must also be provided in the manuscript.

Field-specific reporting

Please select the one below that is the best fit for your research. If you are not sure, read the appropriate sections before making your selection.

- [x] Life sciences
- [ ] Behavioural & social sciences
- [ ] Ecological, evolutionary & environmental sciences

For a reference copy of the document with all sections, see nature.com/documents/nr-reporting-summary-flat.pdf

Life sciences study design

All studies must disclose on these points even when the disclosure is negative.

Sample size	The sample size was determined by the number of collected phage-host interaction datapoints from Beamud et al (Cell Reports, 2023) and from Ferriol-Gonzalez et al. (Biorxiv, 2024), totalling 10,006 interactions of which 274 interactions were confirmed as positive.
Data exclusions	No data were excluded from the analyses, except for phage-host interaction data points for which no genome sequences are available (a necessary prerequisite for data processing and model construction).
Replication	Every spot test in the in vitro validation was repeated in duplicate or triplicate (in case of discrepant results), starting from glycerol stocks at -80°C (for bacterial cultures) or aliquots in liquid broth at -80°C (for phage stocks). Each phage was also tested on its isolation strain at 1:10 and 1:1000 phage dilutions as positive controls. The machine learning model was evaluated using a conventional cross-validation scheme to ensure reproducibility of the results.
Randomization	Not applicable, no comparisons between groups were made in this study.
Blinding	Not applicable, no comparisons between groups were made in this study.

Reporting for specific materials, systems and methods

We require information from authors about some types of materials, experimental systems and methods used in many studies. Here, indicate whether each material, system or method listed is relevant to your study. If you are not sure if a list item applies to your research, read the appropriate section before selecting a response.

Materials & experimental systems

n/a	Involved in the study
☒	☐ Antibodies
☒	☐ Eukaryotic cell lines
☒	☐ Palaeontology and archaeology
☒	☐ Animals and other organisms
☒	☐ Clinical data
☒	☐ Dual use research of concern
☒	☐ Plants

Methods

n/a	Involved in the study
☒	☐ ChIP-seq
☒	☐ Flow cytometry
☒	☐ MRI-based neuroimaging
